# From disruption to remodeling: the evolution and therapeutic prospects of Neuroimmune Regulatory Circuitry after ischemic stroke

**DOI:** 10.3389/fimmu.2025.1702245

**Published:** 2026-01-12

**Authors:** Yikun Gao, Qing Chen, Rui Tao, Wenrui Han, Zhanyong Zhu, Lijuan Gu

**Affiliations:** 1 Department of Plastic Surgery, Renmin Hospital of Wuhan University, Wuhan, Hubei, China; 2 Central Laboratory, Renmin Hospital of Wuhan University, Wuhan, China; 3 Department of Pharmacy, Renmin Hospital of Wuhan University, Wuhan, Hubei, China

**Keywords:** ischemic stroke, Neuroimmune Regulatory Circuitry, neuroimmunology, sympathetic nervous system, vagus nerve

## Abstract

Ischemic stroke (IS) is a leading cause of death and long-term disability globally, and the efficacy of current reperfusion therapies is limited, highlighting a significant unmet clinical need. This review reconceptualizes IS not as a mere focal brain injury but as a systemic disease driven by the catastrophic collapse of the Neuroimmune Regulatory Circuitry. This sophisticated network, normally responsible for maintaining homeostasis, undergoes a multi-level failure after stroke, beginning with pathological sensory input and culminating in a dysregulated efferent response characterized by sustained sympathetic hyperactivity and Hypothalamic-Pituitary-Adrenal (HPA) axis dysfunction. These aberrant neural commands pathologically alter the phenotype and function of peripheral immune cells, leading to a profound immune imbalance: emergency hematopoiesis generates primed, pro-inflammatory myeloid cells, while the lymphoid lineage suffers massive depletion through apoptosis and sequestration, causing severe lymphopenia. This framework unifies seemingly disparate post-stroke complications—such as Stroke-Induced Immunosuppression (SIIS) and subsequent infections, long-term cardiovascular events fueled by chronic inflammation, and cognitive decline driven by persistent neuroinflammation—as predictable outcomes of this circuitry failure. Consequently, this review argues for a paradigm shift away from single-target therapies towards an “integrative and sequential” approach to treatment. Future strategies should aim to recalibrate this entire circuit, leveraging biomarkers to overcome patient heterogeneity and applying temporally-dependent interventions that inhibit acute injury while promoting chronic repair. This provides a more rational foundation for developing effective neuroprotective and restorative therapies for stroke patients.

## 1 Introduction

Ischemic stroke (IS) is one of the leading causes of death and long-term neurological disability worldwide, imposing a heavy socioeconomic and healthcare burden (1). The cornerstone of current treatment, revascularization therapies centered on intravenous thrombolysis and mechanical thrombectomy, has made significant progress in improving outcomes for some patients (2). However, their application is constrained by a narrow therapeutic time window, strict eligibility criteria, and the potential risk of hemorrhagic transformation (3, 4). Consequently, a majority of patients do not benefit from reperfusion therapies. Even in those who achieve successful revascularization, subsequent challenges such as ischemia/reperfusion injury, neuroinflammation, neuronal apoptosis, and long-term cognitive and emotional impairments remain formidable (5). This highlights a substantial unmet clinical need for therapies that promote neuroprotection and functional recovery.

The traditional pathophysiological view of IS focused primarily on localized cerebral ischemia and energy failure directly caused by vascular occlusion. However, research over the past two decades has profoundly expanded our understanding of this disease, driving a paradigm shift from viewing it as a focal brain injury to a systemic disease (6). The acute ischemic core is not merely an endpoint of neuronal necrosis but also a potent source of biological signals. Endogenous molecules released from numerous necrotic cells, such as High-Mobility Group Box 1 (HMGB1), ATP, and mitochondrial DNA, act as Damage-Associated Molecular Patterns (DAMPs) and escape into the peripheral circulation through the compromised Blood-Brain Barrier (BBB) (7, 8). These signaling molecules rapidly activate the peripheral innate and adaptive immune systems, triggering an acute and dysregulated systemic inflammatory response.

Compounding this complexity, this initial “inflammatory storm” is followed by a rapid transition into a profound and persistent state of systemic immunosuppression, known as Stroke-Induced Immunosuppression (SIIS) (9–12). This comprehensive downregulation of immune function significantly increases the patient’s risk of opportunistic infections, particularly Stroke-Associated Pneumonia (SAP) (13). SAP is a key risk factor contributing to early mortality, prolonged hospitalization, and poor long-term prognosis in stroke patients (14). Therefore, the post-stroke pathological progression is essentially the result of a bidirectional, dynamic, and imbalanced interaction between the Central Nervous System (CNS) injury and the systemic immune system.

To systematically integrate this complexity, we propose a unifying “Neuroimmune Regulatory Circuitry” framework. Under physiological conditions, this circuitry maintains immune homeostasis through a closed-loop network comprising afferent sensory pathways (detecting peripheral immune signals) (15), central integration hubs (hypothalamus, brainstem) (16), and efferent response arms (HPA axis and autonomic nervous system) (17). In stroke, catastrophic failure of this circuitry—from pathological sensory input and disruption of integration nodes to dysregulated efferent outputs—drives the transition from acute neuroinflammation to systemic immunosuppression, linking seemingly disparate complications such as infection, cardiovascular events, and cognitive decline.

Using this framework, we systematically examine how IS disrupts each component of the circuitry and explore therapeutic strategies for its recalibration.

## 2 The operation of the physiological Neuroimmune Regulatory Circuitry

**Figure 1:**
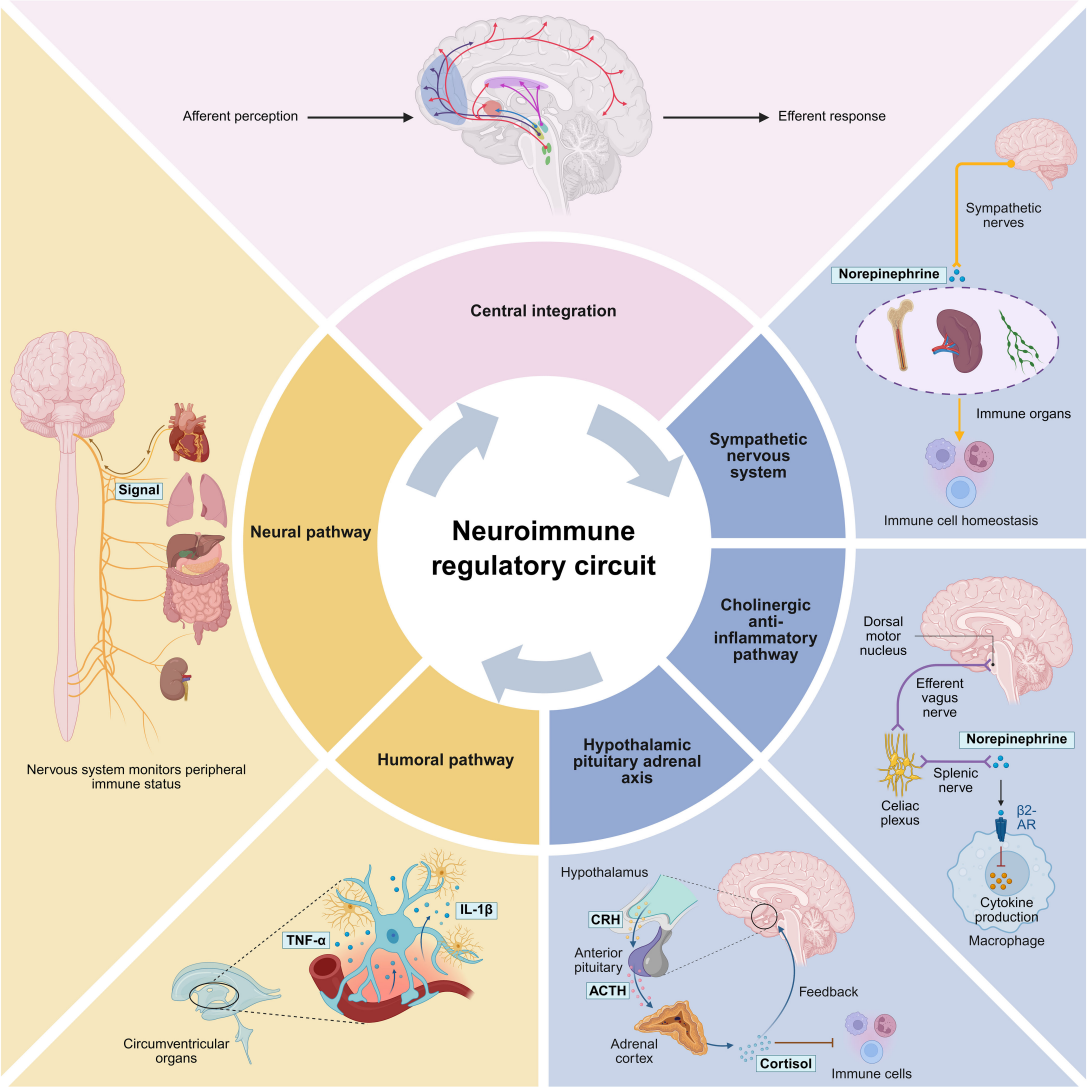
Physiological Neuroimmune Regulatory Circuitry.

Physiological homeostasis relies on a tightly coupled, multi-layered network connecting the nervous and immune systems. This sophisticated interface ensures that immune responses are precisely initiated to clear pathogens and resolutely terminated to prevent autoimmunity. Here, we dissect the cellular and molecular architecture of this maintenance system, tracing the signal flow from peripheral sensing to central processing and effector execution (**Figure 1**).

### 2.1 The afferent sensory arm

The CNS relies on a set of parallel and complementary information input systems to monitor the peripheral immune status, ensuring that any immune perturbation is promptly detected. The Vagus Nerve is a key component of this pathway. The cell bodies of its afferent neurons are located in the nodose ganglion, and experimental studies have demonstrated that the chemoreceptors on their fiber terminals can directly detect immune molecules in the circulatory system. Specifically, it has been experimentally confirmed that these neurons or their adjacent satellite glial cells express Pattern Recognition Receptors (PRRs) and cytokines receptors, such as Toll-like receptor 4 (TLR4) and the Interleukin-1 receptor (IL-1R) (18). Upon encountering pathogen-associated molecules like lipopolysaccharide (LPS) or endogenous cytokines, electrophysiological recordings have shown that these receptors are activated, triggering depolarization of the nerve endings and efficiently transducing chemical signals into action potentials (19). This neural signal is transmitted to the brainstem at millisecond speeds along the vagal pathway, constituting the most direct and rapid route for the brain to perceive acute, systemic inflammation.

For inflammation confined to specific tissues, perception is primarily handled by spinal sensory neurons that innervate the area. The cell bodies of these neurons are in the Dorsal Root Ganglia (DRG). The terminals of their nociceptive fibers (mainly unmyelinated C-fibers and thinly myelinated Aδ-fibers) express a range of receptors that recognize changes in the inflammatory microenvironment. For example, experimental activation assays have demonstrated that Transient Receptor Potential Vanilloid 1 (TRPV1) can be activated by tissue acidosis resulting from inflammation, while ATP-induced activation of P2X3 receptors has been well validated in DRG neuron preparations (20). Furthermore, the direct activation of these neurons by bradykinin and prostaglandins has been repeatedly confirmed in ex vivo preparations. Thus, the spinal afferent pathway enables the precise localization and nociceptive assessment of local inflammation, transmitting this information to the CNS and providing the spatial basis for generating localized neuro-regulatory responses (21).

Additionally, specific anatomical locations of the BBB, the circumventricular organs (CVOs), such as the area postrema and median eminence, have been experimentally shown to permit direct entry of circulating IL-1β, TNF-α, and leptin into the brain parenchyma (22). Here, they can directly bind to receptors on neurons and glial cells, forming a non-neuronal, direct humoral pathway for immune signals to reach the CNS. Microglia and astrocytes, especially those surrounding blood vessels, are the CNS’s resident immune cells and key signal integration units. They express a full suite of PRRs and can keenly respond to trace immune signals transmitted through the aforementioned pathways. Upon activation, glial cells not only release neuromodulatory molecules like prostaglandin E2 (PGE2) and nitric oxide (NO) but also synthesize and release pro-inflammatory cytokines like IL-1β. This forms a positive feedback loop that locally amplifies the initial immune signal within the brain and propagates it to a broader neuronal network, thereby participating in the regulation of central pathophysiological responses such as fever, anorexia, and “sickness behavior” (23).

Beyond the classic neural and humoral pathways, the gut and its vast symbiotic microbiome constitute a fundamental component of the afferent sensory arm of the Neuroimmune Regulatory Circuitry (24). The gut-brain axis provides a continuous and rich stream of information to the CNS about the body’s peripheral state through multiple mechanisms (25). First, the gut microbiota is a major source of numerous circulating immunomodulatory molecules. Short-Chain Fatty Acids (SCFAs), such as butyrate, propionate, and acetate, produced by the fermentation of dietary fiber, can cross the BBB and directly influence CNS functions, including the regulation of neuroinflammation (26). Second, animal models have clearly shown that microbial metabolites and cellular components can affect the permeability of the intestinal barrier. In a state of leaky gut, these molecules enter the circulation, becoming potent systemic inflammatory signals perceived by the CNS. Finally, the gut microbiota can “dialogue” directly with the brain via the vagus nerve, which is the most direct neural pathway connecting the gut and the brainstem (26). Therefore, the gut-brain axis is not an external regulator but a core sensory organ of the neuroimmune circuitry, and its integrity is crucial for maintaining physiological homeostasis.

Beyond microbial metabolites and vagal signaling, post-stroke dysbiosis can compromise the intestinal barrier, leading to bacterial translocation into the circulation and peripheral organs. This phenomenon has been documented in experimental stroke models, where intestinal bacteria can be detected in blood, spleen, and lymph nodes after stroke (27–29). Such bacterial translocation not only amplifies systemic inflammation but also directly contributes to post-stroke infections, representing another critical link between gut dysfunction and immune dysregulation (30).

### 2.2 The central integration hub

Incoming immune information is not simply relayed within the CNS, instead, it undergoes multi-level, complex processing and integration from the brainstem to the cortex to formulate adaptive regulatory decisions. The Nucleus of the Solitary Tract (NTS) is the main terminal nucleus for vagal afferent fibers and the primary gateway for all visceral sensory information entering the brain (31). Neuroanatomical tracing and electrophysiological studies have demonstrated that the NTS has a fine-grained subnuclear structure and functional topology, allowing it to perform initial decoding and classification of incoming signals and relay the information along multiple pathways: one part is transmitted to adjacent brainstem autonomic nuclei, such as the Dorsal Motor Nucleus of the Vagus (DMNX), for rapid reflex responses, while another part is projected to higher centers for further processing (32).

The hypothalamus, particularly its Paraventricular Nucleus (PVN), is the highest-order center for integrating immune, endocrine, and autonomic responses (33). Converging anatomical, electrophysiological, and neuroendocrine experiments have shown that immune signals from the NTS are integrated with inputs from the limbic system and the cortex. The PVN contains functionally distinct neuronal populations: some are neuroendocrine cells that regulate the HPA axis, while others are premotor neurons that control autonomic output. This architecture allows the hypothalamus to coordinately initiate systemic stress and immunoregulatory responses based on the integrated information (34).

### 2.3 The efferent response arm

The CNS’s regulatory commands are ultimately executed through two major efferent systems, the ANS and the neuroendocrine system, to exert precise, long-range control over distant immune organs. Under physiological conditions, postganglionic fibers of the SNS, such as the splenic nerve, extend into the parenchyma of immune organs like the bone marrow, spleen, and lymph nodes (35). Neuroanatomical and ultrastructural studies have demonstrated that their terminals form bead-like axonal enlargements known as varicosities, which establish close, functional contacts with immune and related cells, such as T cells, macrophages, and bone marrow stromal cells. This structure is termed the “neuro-immune synapse” (36). At this junction, experimental observations have confirmed that sympathetic nerves release Norepinephrine (NE) at a basal tone in a manner analogous to “wired communication” (37). By acting on β2-adrenergic receptors on the surface of immune cells, *in vivo* and ex vivo studies have shown that NE finely tunes lymphocyte recirculation and homing, as well as the balance between quiescence and mobilization of hematopoietic stem cells in the bone marrow. This is fundamental to maintaining the immune system’s daily surveillance functions and homeostatic reserve (38). The CAP is the core mechanism by which the parasympathetic nervous system suppresses inflammation. Its efferent signal originates in the DMNX in the brainstem, travels via the vagus nerve to the celiac ganglion, and then adrenergic splenic sympathetic nerves innervate the spleen (39). Regarding its downstream cellular and molecular mechanisms, recent studies have fundamentally revised the classic model. Current mainstream experimental evidence indicates that NE released from splenic nerve terminals directly acts on β2-adrenergic receptors (β2-AR) expressed on the surface of splenic macrophages, thereby directly inhibiting the production of TNF (40). This T-cell-independent, direct pathway is considered the core mechanism by which the CAP exerts its primary anti-inflammatory effects. In contrast, the classic “relay model” is now considered a historical and incomplete model. This model proposed that NE first acted on a special class of acetylcholine (ACh)-synthesizing T cells (ChAT^+^ T cells), which then served as a relay station to release ACh, inhibiting macrophage function via the α7 Nicotinic Acetylcholine Receptor (α7nAChR) (41). Although activation of α7nAChR does indeed inhibit the NOD-like receptor thermal protein domain associated protein 3 (NLRP3) inflammasome and the NF-κB pathway, new comparative studies suggest that the NE-mediated direct pathway plays the dominant role in physiologically Vagus Nerve Stimulation (VNS) induced inflammation suppression.

The HPA axis is the most classic endocrine efferent pathway for responding to inflammation and stress. Driven by Corticotropin-Releasing hormone (CRH) from the hypothalamic PVN, the anterior pituitary secretes Adrenocorticotropic hormone (ACTH), which in turn stimulates the adrenal cortex to synthesize and release Glucocorticoids (GCs) (42). GCs travel through the bloodstream to the entire body and bind to the Glucocorticoid receptor (GR) within immune cells. After the activated GR complex enters the nucleus, it exerts powerful anti-inflammatory and immunosuppressive effects through two main mechanisms: (1) Transrepression: It interacts with pro-inflammatory transcription factors like NF-κB and AP-1 to inhibit their transcriptional activity; (2) Transactivation: It directly binds to Glucocorticoid response elements (GREs) on DNA to upregulate the expression of anti-inflammatory genes (43).

In summary, the Neuroimmune Regulatory Circuitry in its physiological state is a multi-layered, multi-pathway, and functionally sophisticated network. Through a continuous “Perception-Integration-Response” closed loop, it ensures the dynamic equilibrium of the immune system, providing a solid guarantee for the health of the organism.

## 3 Disruption of the Neuroimmune Regulatory Circuitry after IS

**Figure 2:**
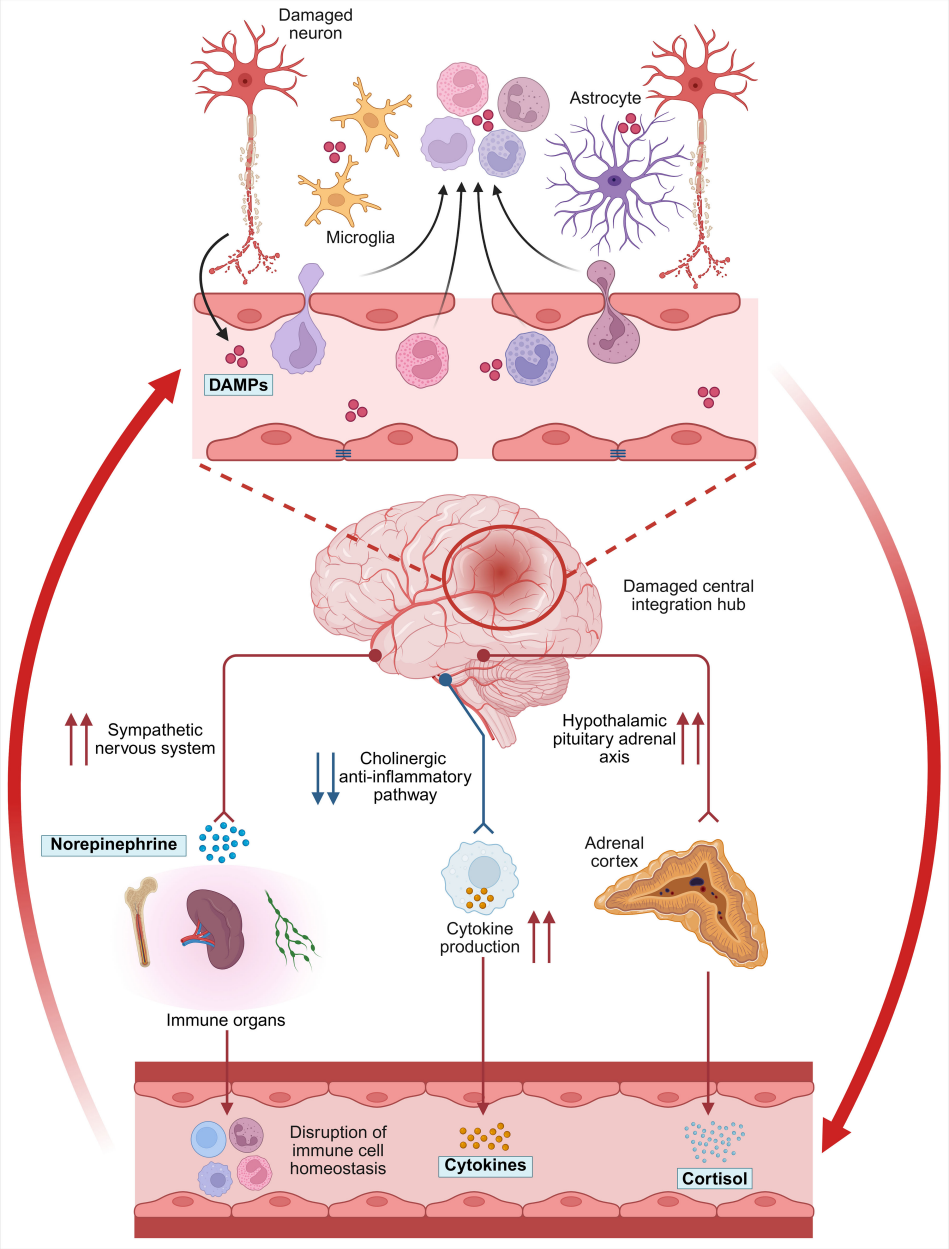
Disruption of the Neuroimmune Regulatory Circuitry after Ischemic Stroke (IS).

The impact of IS on the Neuroimmune Regulatory Circuitry is not a simple functional deviation but rather a catastrophic event that simultaneously triggers pathological signal input and structural damage at all critical nodes, including perception, integration, and response. This series of events transforms the entire system from a sophisticated feedback network for maintaining homeostasis into a self-amplifying cascade that drives pathological progression (**Figure 2**).

### 3.1 Functional failure of the perception and integration arms

In the context of IS, the applicability of classic afferent sensing pathways requires clarification. Unlike peripheral inflammatory insults where vagal and spinal afferents are primary sensors, the initial immune signal in stroke originates centrally—from neuronal necrosis, DAMP release, and BBB disruption within the CNS itself. Therefore, early neuroimmune dysregulation in IS likely dominated by disruption of central integration hubs (PVN, brainstem) and CNS-intrinsic DAMP signaling, with peripheral afferent sensing playing a secondary role. Classic afferent pathways may become more relevant for sensing subsequent systemic inflammation or peripheral complications (e.g., pneumonia).

In the ischemic core, the necrotic death of neurons and glial cells caused by severe energy failure constitutes the initiating event of the pathological process (44). Unlike physiological programmed apoptosis, necrosis leads to the rupture of the cell membrane, releasing a massive amount of normally intracellular molecules into the tissue interstitium. These endogenous molecules, including HMGB1, members of the S100 protein family, ATP, and mitochondrial DNA, constitute DAMPs (7, 45, 46). Concurrently, the integrity of the BBB is compromised after stroke, which not only allows peripheral immune cells to enter the brain tissue but also creates a conduit for CNS-derived DAMPs to leak into the systemic circulation (47). This flood of DAMPs from the CNS into the bloodstream hijacks physiological immune-sensing pathways, initiating a systemic and inappropriate immune response. Within the brain parenchyma, DAMPs trigger a fulminant neuroinflammatory response by activating PRRs on microglia and astrocytes (48). This intense inflammatory environment within the brain provides a sustained and abnormal stimulus to the central integration nodes, overwhelming their function with pathological signals.

The anatomical location of the stroke lesion has a decisive impact on the disruption of the Neuroimmune Regulatory Circuitry. When the ischemic injury affects key brain regions responsible for autonomic regulation, the integration arm suffers structural damage. Both clinical and basic research have shown that injury to the right insular cortex, a high-order regulatory center for the sympathetic nervous system, is closely associated with elevated plasma catecholamine levels and an increased frequency of post-stroke infections (49). However, it should be noted that infarct volume itself is often a stronger correlate of SIIS and infections. The insular cortex association may be partially confounded by lesion size and overall stroke severity, and further studies controlling for these factors are needed to establish its independent contribution. Similarly, if the ischemic lesion or subsequent cerebral edema directly damages the hypothalamic PVN or the brainstem, it will directly cripple the primary control centers of the HPA axis and autonomic reflexes, leading to their functional paralysis (50). Even if the integration hubs are not directly damaged, their function is still severely suppressed. Supraphysiological cortisol concentrations induced by stroke-related stress, excitotoxicity in the ischemic penumbra, and peri-infarct depolarizations can all cause widespread functional disturbances at the neuronal network level. This renders structurally intact integration hubs incapable of processing incoming signals effectively and accurately, leading to the output of erroneous regulatory commands (51, 52).

### 3.2 Dysregulation of the efferent response

The dysregulated central output ultimately leads to a severe imbalance in the efferent response arm, its core feature being a hyperactivity of the sympathetic nervous system and a failure of the parasympathetic-mediated anti-inflammatory pathway.

First, the activity of the SNS exhibits an acute and sustained hyperactivity, leading to a massive systemic release of NE. In the spleen, NE binds to β2AR on the surface of T lymphocytes, activating the downstream cAMP/PKA signaling pathway (53, 54). The overactivation of this pathway has two major consequences: on one hand, it profoundly suppresses T cell proliferation and the production of cytokines like Interferon-γ (IFN-γ) by phosphorylating transcription factors such as CREB and inhibiting proximal TCR signaling molecules, inducing a state of functional paralysis (55, 56), on the other hand, it may contribute to T cell apoptosis via upregulation of pro-apoptotic proteins from the BH3-only family, such as Bim and PUMA, activating the Caspase-3-mediated mitochondrial apoptosis pathway (57). While β-blockade studies show reduced lymphopenia, the extent to which this reflects direct β2-adrenergic effects on T cells versus indirect mechanisms (e.g., HPA-mediated glucocorticoid action, altered stromal signals, trafficking changes) remains to be fully established. The relative contribution of each pathway requires further lineage-specific investigation. The net result is a massive depletion of effector T cells, such as CD4+ and CD8+ T cells, in the spleen, leading to rapid splenic atrophy. Meanwhile, the relative proportion of immunosuppressive Regulatory T cells (Tregs) increases, collectively establishing profound cellular immunosuppression (58).

Beyond splenic sympathetic innervation, SNS activation can also act directly on the adrenal medulla to modulate circulating catecholamines and glucocorticoids. In chronic stress models, increased SNS drive enhances basal and rapid corticosterone production via adrenal mechanisms, partially independent of early ACTH dynamics (59). Whether a similar SNS-adrenal route operates in acute ischemic stroke, alongside splenic innervation and HPA activation, warrants further investigation.

This intense sympathetic drive also acts on the bone marrow (60). By binding to β3AR on stromal cells within the bone marrow microenvironment, NE potently suppresses the production of the key chemokine CXCL12 (61). CXCL12 is the primary retention signal that anchors Hematopoietic Stem/Progenitor Cells (HSPCs) in their quiescent state within the bone marrow niche. Its downregulation leads to the mobilization of vast numbers of HSPCs into the proliferative pool. These emergently mobilized myeloid cells are often phenotypically and functionally immature and, upon release into the bloodstream, become the primary cellular effectors of subsequent tissue damage and inflammation (62).

In summary, by inducing multidimensional disruptions across the perception, integration, and response arms, ischemic stroke transforms a sophisticated regulatory circuit designed for homeostasis into a key driver of pathological progression, providing a clear mechanistic framework for understanding its complex clinical complications.

## 4 Pathological reprogramming of immune cell function under dysregulated circuitry commands

The disruption of the Neuroimmune Regulatory Circuitry after IS manifests as specific cellular pathologies through the pathological reprogramming of peripheral immune cell functions. This reprogramming process profoundly affects both the myeloid and lymphoid lineages, altering their generation, phenotype, migration, and effector functions.

### 4.1 Myeloid cells under dysregulated commands

Emergency hematopoiesis, mediated by the runaway sympathetic drive and a dysregulated systemic factor environment, is not merely a surge in cell numbers but a fundamental alteration in their intrinsic quality and functional potential (63). As a direct product of emergency granulopoiesis, circulating neutrophils exhibit significant features of immaturity, such as a nuclear left shift. More importantly, they exist in a “primed” state: sustained exposure to high concentrations of catecholamines and Granulocyte Colony Stimulating Factor (G-CSF) in the bone marrow leads to the pre-assembly of their internal Reactive Oxygen Species (ROS)-generating enzymes (e.g., NADPH oxidase) and a significantly lower activation threshold for NETosis (64, 65). This indicates that neutrophils mobilized by the dysregulated circuitry are programmed from their inception into a more destructive cellular state.

Another consequence of the dysregulated neural circuitry on bone marrow lineage differentiation is the selective expansion of the classical (pro-inflammatory) monocyte subset (Ly6C^high^ in mice) (60). This cell lineage skewing, dictated by dysregulated commands, fills the peripheral circulation with monocytes that highly express the chemokine receptor CCR2. These cells are not only exceptionally sensitive to chemoattractant signals from the brain but are also intrinsically poised with a greater potential to differentiate into pro-inflammatory M1-like macrophages (66).

The migration of these pathologically reprogrammed myeloid cells into the ischemic brain tissue, in its scale and efficiency, is a direct reflection of the failed control of the central local microenvironment. The release of DAMPs and the eruption of neuroinflammation within the brain are direct manifestations of the loss of control in the central “perception” and “integration” arms (67). This uncontrolled state leads to the over-activation of cerebral microvascular endothelial cells, which sequentially upregulate E/P-selectins and Intercellular Cell Adhesion Molecule-1 (ICAM-1)/Vascular Cell Adhesion Molecule-1 (VCAM-1), thereby initiating the leukocyte adhesion cascade from capture and rolling to firm adhesion (68, 69). Building on this adhesion process, a chemokine gradient produced by activated glial cells and neurons in the brain guides leukocytes across the endothelium (70). Crucially, the intensity and persistence of this chemoattractant gradient are a direct consequence of the failure of anti-inflammatory mechanisms.

Upon entering the brain parenchyma, the behavioral choices of immune cells are further distorted by dysregulated local microenvironmental signals. Infiltrating macrophages are overwhelmingly polarized towards a pro-inflammatory M1-like phenotype during the acute phase (71). This overwhelming bias profoundly reflects the functional deficit of vagal nerve inhibition. In the absence of effective constraint from α7nAChR signaling, the response of macrophages to signals like DAMPs and IFN-γ is excessively amplified, leading to the uninhibited release of their NF-κB and other pro-inflammatory pathways and the production of an overabundance of neurotoxic mediators (72).

The failure of neural repair after stroke may be linked to the long-term, chronic dysregulation of the neural circuitry. A promising hypothesis is that a sustained state of stress (e.g., HPA axis rhythm disruption and low-grade sympathetic activation) creates a microenvironment that is inhibitory to tissue repair. This “chronic alarm” state, maintained by long-term circuit dysregulation, may impede the effective transformation of macrophages towards an M2-like phenotype by suppressing key pro-reparative signaling pathways like IL-4/Signal Transducer and Activator of Transcription 6 (STAT6), thereby stalling the neural repair process (73).

### 4.2 Lymphoid cells under dysregulated commands

In stark contrast to the expansion of myeloid cells, lymphoid cells undergo a sharp decline in number and a profound functional remodeling after stroke, which is also a direct consequence of neural circuit dysregulation. The prominent post-stroke peripheral lymphopenia is a systemic manifestation of the synergistic effects of dysregulated efferent pathways (74). While the precise cellular and molecular mechanisms remain incompletely defined, current evidence suggests multiple contributing pathways. First, sympathetic overactivation may contribute to lymphocyte apoptosis in secondary lymphoid organs (spleen, lymph nodes), as β-blockade studies show reduced lymphopenia (75). second, the hypercortisolemia resulting from HPA axis dysfunction induces widespread lymphocyte apoptosis by activating the glucocorticoid receptor (76). However, the extent to which sympathetic effects reflect direct β2-adrenergic signaling to T cells versus indirect mechanisms—such as glucocorticoid-mediated actions, altered stromal cell signals, or trafficking changes—requires further lineage-specific investigation. The net result is a massive depletion of effector lymphocytes through the combined action of these pathways.

Furthermore, lymphocyte trafficking is governed by the Sphingosine-1-phosphate (S1P)/S1P receptor 1 (S1PR1) signaling axis. Under physiological conditions, lymphoid organs maintain low S1P concentrations while blood and lymph exhibit high S1P levels, creating a chemotactic gradient (77). Lymphocytes expressing S1PR1 sense this gradient and exit into circulation (78).

After stroke, this system undergoes complex alterations. First, plasma S1P levels are dysregulated and correlate with stroke severity and outcome (79). Second, atypical S1P metabolites and analogues accumulate, which have pathological implications including increased risk of hemorrhagic transformation (80) and altered immune cell behavior (81). Notably, exposure of infiltrating immune cells (such as cytotoxic T cells) to these atypical S1P analogues drives their pathological reprogramming, enhancing IFNγ production that subsequently activates microglia and amplifies neuroinflammation (82). Third, S1P signaling influences lymphocyte trafficking: disruption of the S1P gradient or S1PR1 downregulation can impair lymphocyte egress (83, 84). In certain contexts, such as CNS tumors, S1P-mediated sequestration of T cells in bone marrow has been reported (85), though whether similar sequestration occurs in stroke-related lymphopenia remains unclear and requires direct histological evidence.

In summary, S1P system dysregulation may contribute to post-stroke lymphopenia through multiple mechanisms—altered gradients, receptor modulation, and potentially sequestration—but its relative importance compared to apoptosis and HPA/SNS-mediated depletion requires further clarification.

Beyond the reduction in numbers, a small quantity of T cells can still infiltrate the post-stroke brain and play a dual role at different stages of the disease. At the molecular level, infiltrating T cells undergo transcriptomic and epigenetic reprogramming after stroke. Recent studies have demonstrated that stroke-recruited T cells, including Tregs, exhibit distinct gene expression profiles compared to circulating or lymphoid tissue T cells (86). For instance, CNS-infiltrating Tregs upregulate tissue-residency and immunosuppressive markers, enhancing their local regulatory function (86). Similarly, effector T cells can be reprogrammed by the post-stroke CNS microenvironment—including exposure to atypical sphingolipids (82), hypoxia, and cytokines—to adopt pathogenic phenotypes characterized by enhanced IFNγ or IL-17 production (87). This reprogramming is not merely a functional shift but involves stable epigenetic modifications that may persist and influence long-term outcomes. In the subacute and chronic phases, Treg cells are recruited to the brain, where they can suppress microglial overactivation and promote neural repair by secreting anti-inflammatory cytokines like IL-10 (88). Conversely, the infiltration of pro-inflammatory T cell subsets such as Th1 and Th17 cells may exacerbate BBB damage and neuronal injury by releasing cytokines like IFN-γ and IL-17 (89).

The B cell system also undergoes profound dysregulation and functional reprogramming, and its functional heterogeneity is rooted in multiple factors, including B cell subsets, anatomical localization, and patient context (90). In the subacute and chronic phases of stroke, B cells exhibit a distinctly detrimental side (91). Some B cells can infiltrate the damaged brain tissue, aggregating to form “ectopic lymphoid structures”. These structures provide a microenvironment for B cell survival and differentiation into plasma cells, which produce IgG and IgA against CNS self-antigens (92). This B cell-mediated autoimmune response is considered a key mechanism for delayed post-stroke cognitive decline (92). At the same time, specific B cell subsets also play an indispensable protective role during the post-stroke recovery phase (91). These beneficial effects are mainly attributed to regulatory B cells, which secrete IL-10. These B cells have been found to migrate to brain regions distant from the infarct core that are closely related to neural repair (such as the hippocampus), promoting neurogenesis and functional recovery through their supportive actions (93). Recent studies have found that a subset known as Age-associated B-cells (ABCs) plays a role in stroke recovery (90). Given that ABCs are closely linked to autoimmunity in other disease contexts, this finding suggests that the patient’s age may be a key variable determining the functional trajectory of B cells (94).

In conclusion, the dysregulated Neuroimmune Regulatory Circuitry after stroke is the prime driver behind the functional reprogramming of the immune system. It not only drives the emergency hematopoiesis and pro-inflammatory “priming” of myeloid cells but also delivers a dual blow to the lymphoid system: first, through SNS and HPA axis overactivation, it triggers massive peripheral lymphocyte depletion characterized by apoptosis and altered trafficking; second, it distorts the function of T and B cells that infiltrate the CNS, causing them to play starkly different dual roles during the course of the disease. This immune imbalance, composed of myeloid cell overactivation and lymphoid lineage suppression, is the key pathological basis of SIIS and significantly increases the risk of secondary infections in patients.

## 5 From neuroimmune circuitry dysregulation to clinical syndromes in patients

Following an IS event, the Neuroimmune Regulatory Circuitry undergoes a catastrophic functional collapse at multiple levels—perception, integration, and response—which in turn drives the reprogramming of downstream immune cell phenotypes and functions. This chapter aims to project these pathophysiological processes onto recognizable, diagnosable, and predictable clinical syndromes. These complications are not isolated clinical events but rather the inevitable outcomes of circuitry dysregulation at the organismal level, and they collectively determine the patient’s final prognosis.

### 5.1 Post-stroke infection

Post-stroke infection, particularly SAP, is one of the most common and fatal post-stroke complications. It constitutes the most direct clinical manifestation of the immunosuppression caused by circuitry dysregulation (95). The fundamental pathophysiological basis of post-stroke infection is SIIS. As previously described, the core drivers of SIIS originate from SNS overactivation and HPA axis dysfunction. These two dysregulated efferent pathways, through mechanisms such as β2AR-mediated apoptosis and hypercortisolemia, collectively lead to a drastic depletion in the number and a profound paralysis in the function of peripheral blood lymphocytes, especially the T cells that serve critical effector functions.

This profound cellular immunodeficiency severely weakens the host’s ability to monitor and clear opportunistic pathogens. The loss of effector T cells, including Th1 and Th17 subsets, contributes to increased susceptibility to infections. While Th1 responses are classically associated with viral and intracellular pathogen defense, the broader loss of T cell-mediated immunity—including impaired cytokine production, reduced macrophage activation, and defective antibody responses—collectively compromises defense against common respiratory bacteria like Streptococcus pneumoniae and Staphylococcus aureus. Additionally, the relative preservation or expansion of immunosuppressive Tregs further tilts the balance toward infection susceptibility (10).

Furthermore, this immune deficiency synergizes with other neurogenic dysfunctions. An impaired swallowing reflex caused by autonomic dysfunction significantly increases the risk of aspiration (96). Simultaneously, reduced gastrointestinal motility may promote the translocation of gut microbiota (97). These factors create conditions for pathogen invasion and colonization, which, when superimposed on the state of immunosuppression, place stroke patients at an extremely high risk of infection. Additionally, post-stroke malnutrition has been identified as a key risk factor for SAP (98). Pathophysiologically, it is closely intertwined with other major risk factors like dysphagia, long-term bed rest, and stroke-induced immunosuppression, forming a vicious cycle that collectively elevates infection risk (99).

### 5.2 Long-term cardiovascular events

During their recovery period, stroke survivors have a significantly higher risk of recurrent stroke or acute myocardial infarction compared to the general population (100). Underlying this phenomenon is a chronic, low-grade systemic inflammatory state ignited and sustained by the dysregulated neural circuitry. The emergency myelopoiesis driven by the dysregulated circuitry continuously releases large numbers of highly pro-inflammatory classical monocytes into the circulation. These monocytes are characterized by high expression of CCR2, making them highly prone to being chemoattracted to pre-existing atherosclerotic plaques within the patient’s body (101). In the plaque microenvironment, they differentiate into pro-inflammatory M1-like macrophages. By releasing matrix metalloproteinases (MMPs) to degrade the plaque’s fibrous cap and producing ROS to oxidize lipids, these cells drive the critical events that transition an atherosclerotic plaque from a stable to an unstable state, which can ultimately lead to plaque erosion or rupture (102).

This mechanism is corroborated by clinical research. Studies using advanced imaging techniques like 18F-FDG PET/CT have found that the inflammatory metabolic activity of aortic and carotid artery plaques is significantly increased in post-stroke patients (103). Concurrently, large-scale epidemiological studies have confirmed that a sustained elevation of inflammatory markers such as C-reactive protein and IL-6 in post-stroke plasma is an independent risk factor for future cardiovascular events (104).

### 5.3 Post-stroke cognitive impairment

PSCI is a severe sequela that affects patients’ quality of life. Its development is not only related to the location and volume of the initial infarct but is also closely linked to a persistent and unresolved state of chronic neuroinflammation (105). The core mechanism of PSCI can be attributed to the long-term, chronic dysregulation of the Neuroimmune Regulatory Circuitry. In a brain microenvironment lacking effective anti-inflammatory and pro-reparative signals, chronically-activated, M1-like microglia/macrophages become long-term disruptors of neuronal network function (106). They cause excessive synaptic pruning and loss by releasing neurotoxic substances (e.g., glutamate, ROS) and activating complement (107). Their damage to Oligodendrocyte Precursor Cells (OPCs) impedes effective remyelination (108). And they fail to efficiently produce essential neurotrophic factors (like BDNF) that support neural plasticity (109). This diffuse and persistent neuroinflammatory state particularly impairs higher-order cognitive functions that rely on the integrity of whole-brain networks, often manifesting clinically as significant declines in executive function, information processing speed, and episodic memory.

### 5.4 Clinical biomarkers indicating neuroimmune circuitry dysregulation

**Figure 3:**
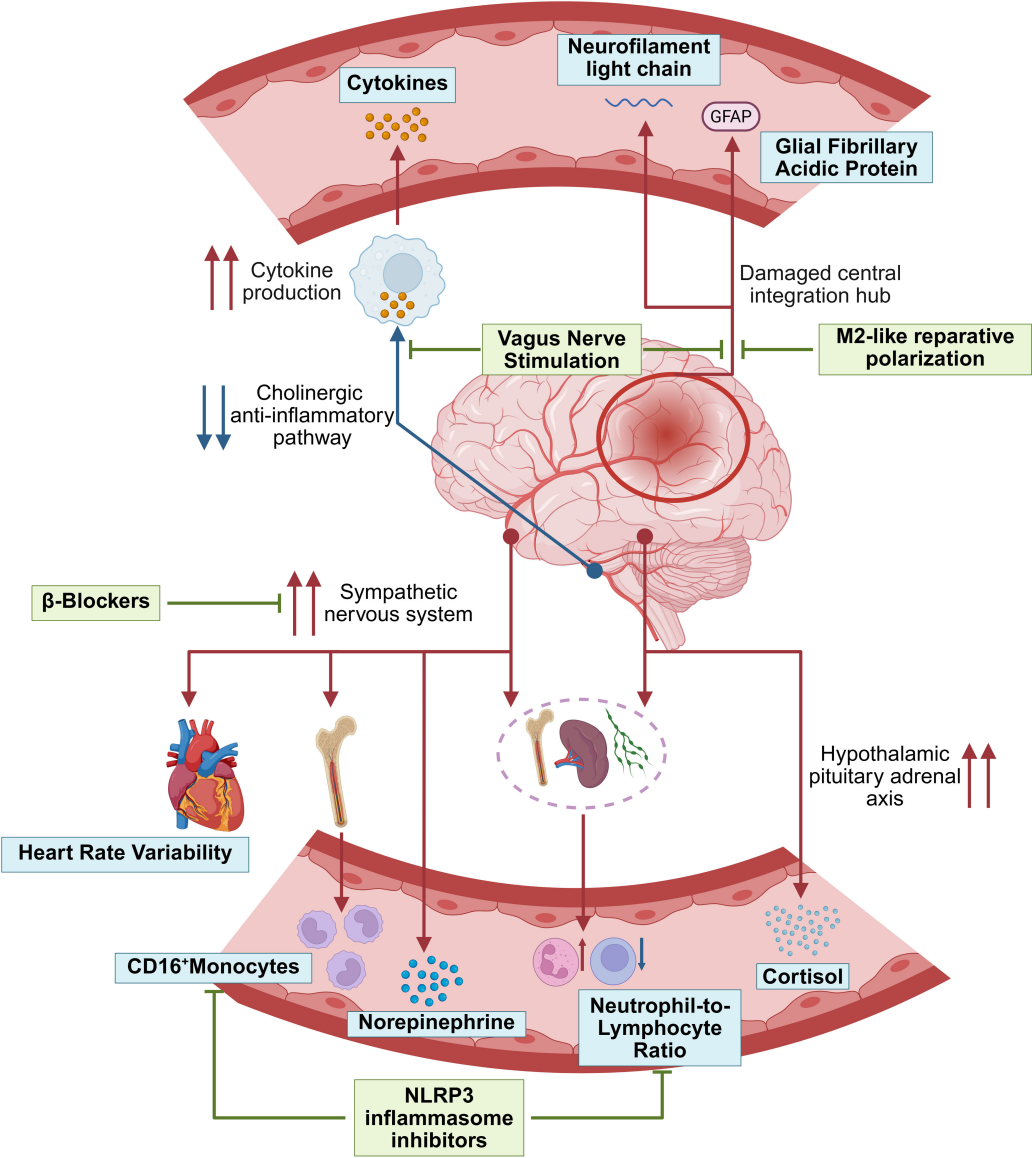
Clinical biomarkers indicating Neuroimmune Regulatory Circuitry dysregulation and therapeutic strategies after IS.

Applying the theoretical framework of Neuroimmune Regulatory Circuitry dysregulation to clinical practice hinges on developing and utilizing biomarkers that can objectively and quantitatively reflect the functional status of each component of this circuit. These markers not only deepen our understanding of the pathophysiology but also have the potential to be used for patient risk stratification, prognostic prediction, and guiding future individualized therapies (**Figure 3**).

#### 5.4.1 Neutrophil-to-lymphocyte ratio

NLR is not a single indicator but a composite biomarker that efficiently reflects the combined force of the two dysregulated efferent pathways. The increase in its numerator (neutrophils) directly reflects SNS-driven emergency myelopoiesis, while the decrease in its denominator (lymphocytes) simultaneously reflects lymphocyte apoptosis mediated by both the SNS and the HPA axis. Therefore, a sharp increase in NLR is a perfect microcosm of the dual effects of circuitry-driven sympathetic hyperactivity and immunosuppression. Due to its simple and inexpensive detection, NLR has become one of the most widely used inflammatory biomarkers in the stroke field (110). Numerous clinical studies have confirmed that acute-phase NLR levels are strongly and positively correlated with National Institute of Health Stroke Scale (NIHSS) score, infarct volume, risk of hemorrhagic transformation, incidence of post-stroke infection, and poor long-term functional outcome, making it a robust independent prognostic predictor (111, 112). However, the specificity of NLR is relatively low, as its level is easily influenced by confounding factors such as co-existing infections and other inflammatory diseases.

#### 5.4.2 Monocyte subset analysis

Through flow cytometry analysis of CD14 and CD16 expression, the expansion of pro-inflammatory classical monocytes (CD14^++^CD16^-^) can be quantified (113). The increase in this specific subset is a direct product of bone marrow lineage skewing under sympathetic command, and changes in its cell count and proportion provide a more specific window for assessing the intensity of emergency myelopoiesis. Studies have shown that an elevated proportion of classical monocytes is associated with higher levels of systemic inflammation (e.g., IL-6) and worse clinical outcomes after IS (114). In theory, it may better predict long-term complications driven by myeloid cells, such as the progression of atherosclerosis. However, flow cytometry analysis is relatively complex and costly, and it is currently used mainly in scientific research rather than as a routine clinical test.

#### 5.4.3 Neurotransmitters and stress hormones

Plasma levels of catecholamines (norepinephrine, epinephrine) and cortisol are not merely markers of the immune response; they are the effector molecules of the Neuroimmune Regulatory Circuitry’s efferent arms themselves (115). Therefore, measuring their concentrations is equivalent to directly quantifying the “power output” of the two key output pathways: the Sympathetic-Adrenal-Medullary (SAM) axis and the HPA axis. A sharp rise in catecholamines and cortisol in the acute phase of stroke is closely associated with stroke severity, cardiac complications (e.g., stress cardiomyopathy, arrhythmias), and short-term mortality (116). However, the secretion of these molecules is pulsatile and follows a diurnal rhythm, and it is highly susceptible to in-hospital environmental factors like pain and stress. This necessitates strict timing and conditions for blood collection, limiting their application as routine biomarkers.

#### 5.4.4 Cytokines profile

Plasma concentrations of certain cytokines, such as the pro-inflammatory IL-6 and the anti-inflammatory IL-10, are a direct reflection of the final “functional output” of downstream immune cells after receiving dysregulated neural commands. High levels of IL-6 are a strong predictor of post-stroke infection, delirium, depression, and poor functional outcome (117). Plasma IL-10 levels are an independent predictor of systemic infection after stroke (118).

#### 5.4.5 Markers of neural injury

Plasma levels of molecules like Neurofilament light chain (NfL) and Glial Fibrillary Acidic Protein (GFAP) primarily reflect the severity of the initial brain injury—that is, the intensity of the “initiating event” that triggers the entire circuitry’s dysregulation (119). Their levels are closely correlated with infarct volume, long-term neurological deficits, and the degree of brain atrophy (120). Analyzing these injury markers in conjunction with immune markers can help distinguish whether an immune response is passively triggered by extensive damage or if a patient’s neuroimmune circuitry is inherently overreactive for a given degree of injury (121).

#### 5.4.6 Heart rate variability

HRV is currently the most mature and reliable non-invasive technology for assessing autonomic nervous function in the clinical setting. By analyzing minute fluctuations in the intervals between heartbeats, it quantifies the regulatory tone of the sympathetic and parasympathetic nerves on the heart (122). Among its components, the high-frequency power is widely recognized as a highly specific surrogate marker for cardiac vagal (parasympathetic) activity (123). Therefore, HRV analysis provides a unique window to directly quantify the functional integrity of the cholinergic anti-inflammatory pathway, the primary anti-inflammatory branch of the Neuroimmune Regulatory Circuitry. A large body of research has consistently shown that a significant reduction in HRV in the acute phase of stroke, especially a decrease in the HF component, is a powerful independent predictor of mortality, severe infection, and poor functional outcome (124, 125).

In conclusion, post-stroke infection, long-term progression of atherosclerosis, and cognitive impairment—clinical challenges that seemingly belong to different domains—can be unified under the theoretical framework proposed in this review as diverse manifestations of the Neuroimmune Regulatory Circuitry’s collapse across different time dimensions and organ systems. More importantly, the emergence of a series of cellular, humoral, and functional biomarkers now enables us to clinically quantify this dysregulated circuitry. This not only profoundly reveals a patient’s prognostic trajectory but also lays a solid theoretical and practical foundation for the future development of targeted therapeutic strategies aimed at “recalibrating” the circuit’s function.

## 6 Therapeutic strategies targeting the recalibration of the neuroimmune regulatory circuitry and future paradigms

Building upon the systematic elucidation in the preceding chapters of the comprehensive collapse of the Neuroimmune Regulatory Circuitry post-stroke, this chapter will focus on the therapeutic rationale of various interventions, key preclinical and clinical evidence, and the real-world challenges they face in translational application. We further propose that to address the complex pathology of stroke, future immunotherapy must evolve from a single-target mindset to a new paradigm based on the circuit’s overall function, emphasizing an “integrative and sequential” approach (**Figure 3**).

### 6.1 Targeting the efferent response arm: direct intervention on peripheral immune output

Directly modulating the dysfunctional autonomic efferent pathways is the most direct and relatively well-studied strategy for intervening in the neuroimmune circuit, aiming to curb erroneous neural commands at their source. The core purpose of VNS is to functionally compensate for the endogenous CAP that has failed due to central and peripheral causes, with the goal of restoring the physiological capacity to suppress systemic inflammation (126). A large body of preclinical research has confirmed that in experimental stroke models, VNS can significantly reduce infarct volume, lower the levels of pro-inflammatory cytokines (e.g., TNF-α, IL-6) in serum and brain tissue, and improve neurological outcomes (127). Its therapeutic potential is markedly pleiotropic: not only does it hold promise for suppressing peripheral inflammation and reducing post-stroke infection risk by activating the CAP, but VNS can also promote neuroplasticity through dopaminergic pathways (128). This has been confirmed in clinical trials where VNS combined with rehabilitation therapy significantly improved upper limb function in chronic stroke patients (129, 130). Despite its great promise, the application of VNS in the stroke field is still in an exploratory phase. Invasive VNS carries surgical risks, while transauricular non-invasive VNS (tVNS) faces challenges in standardizing stimulation sites and ensuring effective activation of the target nerve. Defining the optimal therapeutic window and stimulation parameters for VNS in different stages of stroke is critical for its successful translation (131).

An additional layer of complexity arises from recent experimental findings suggesting that selective β2-adrenergic agonists (e.g., clenbuterol) may reduce post-stroke pneumonia in certain experimental models (75). At first glance, this appears paradoxical, given that excessive SNS activation is a key driver of stroke-induced immunosuppression. This contradiction may reflect the mechanistic differences between physiological SNS activation—which simultaneously and dynamically engages β1-, β2- and α-adrenergic receptors—and the highly selective receptor targeting achieved by pharmacological β2 agonists. Differences in drug timing, dosing, and kinetics compared with endogenous catecholamine surges, as well as cell-type-specific β2AR signaling in neutrophils, macrophages, epithelial cells, and lymphocytes, may likewise contribute. These findings collectively highlight that simple “augmentation” or “inhibition” of adrenergic signaling is unlikely to recapitulate the nuanced physiological control exerted by the autonomic circuitry, reinforcing the rationale for interventions acting at the level of the efferent pathways rather than isolated receptors.

Despite these mechanistic complexities and the theoretical limitations of receptor-targeted therapies, the use of β-blockers to directly counter the series of core pathophysiological consequences driven by SNS hyperactivity—including immunosuppression, emergency myelopoiesis, and cardiovascular toxicity—remains a conceptually attractive intervention strategy (132), albeit one requiring careful evaluation in the clinical context. The clinical evidence for this strategy is fraught with contradictions (133). One historical cohort study found no change in postoperative pneumonia risk among β-blocker users, but a reduced risk of Urinary Tract Infections (UTIs) (134). In contrast, another study concluded the opposite, finding an association between β-blocker use and an increased risk of UTIs or SAP in some stroke patients (133). Regarding the impact on mortality, one study found that in acute IS patients with comorbid tachycardia (heart rate ≥100 bpm), the continued use of β-blockers (i.e., maintaining therapy post-stroke) was associated with a significant reduction in long-term mortality, whereas discontinuation was linked to an increased risk of early mortality (135). The current state of evidence underscores the urgency of conducting large-scale, prospective Randomized Controlled Trials (RCTs). Such trials need to clearly differentiate between types of β-blockers and target specific patient subpopulations (e.g., those with comorbid tachycardia) to ultimately clarify their causal relationship and net clinical benefit.

### 6.2 Targeting immune effector cells: from inhibiting damage to promoting repair

Besides modulating upstream neural signals, directly intervening in the downstream effector cells mobilized by dysregulated commands is another important therapeutic avenue. NLRP3 inflammasome inhibitors (e.g., MCC950) aim to suppress myeloid cell-mediated damage (136). However, the preclinical evidence for this strategy is marked by significant controversy. Although numerous studies using the transient middle cerebral artery occlusion (tMCAO) model have reported positive results (137), a pivotal study using a thromboembolic stroke model, which more closely mimics the clinical reality, found that blocking the NLRP3 pathway, either through genetic knockout or pharmacological inhibition with MCC950, had no effect on the extent of stroke-induced damage (138). This is a pivotal negative finding that directly challenges the generalizability of this therapeutic strategy and highlights how the choice of preclinical model can dramatically alter the efficacy of an immunomodulatory therapy.

Furthermore, one can actively promote M2-like reparative polarization. The core idea of this strategy is to shift from passive anti-inflammation to active pro-reparation, aiming to address the key problem of inefficient neurological recovery after stroke, which is caused by persistent inflammation and insufficient repair signals. This line of inquiry represents the future of stroke immunotherapy. Experimental approaches include: drug repurposing studies using approved nuclear receptor agonists (e.g., the PPARγ agonist pioglitazone) to induce the transformation of macrophages/microglia to an M2 phenotype within the brain (139), direct administration of recombinant cytokines (e.g., IL-4 complexes) to create a pro-reparative microenvironment (140), and exploring cutting-edge cell therapies, such as the intravenous infusion of M2 macrophages differentiated *in vitro (*141). While these strategies are highly promising, they still face numerous challenges, including cell sourcing, *in vivo* tracking, safety, and standardization.

### 6.3 Integrative and sequential therapy: towards a new paradigm in stroke immunotherapy

Given the multi-nodal and dynamic functional collapse of the Neuroimmune Regulatory Circuitry after stroke, the traditional, single-target linear therapeutic mindset has shown its limitations. The failure of numerous clinical trials targeting broad-spectrum anti-inflammation or single cytokines compels us to re-examine the fundamental logic of stroke immunotherapy. We propose here that the future therapeutic paradigm must be built upon the twin pillars of “Integration” and “Temporality” to match the complex pathophysiological process.

Since the post-stroke pathological state results from the synergistic dysregulation of multiple components—including autonomic nerves, neuroendocrine systems, and immune effector cells—an effective intervention should also be multi-targeted. It should aim to act on different functional nodes of the circuit, either simultaneously or sequentially, to produce synergistic or complementary therapeutic effects. First, a Horizontal Integration model can be considered, which involves intervening in parallel pathological pathways within the same time window. A rational theoretical proposal would be to combine a drug aimed at inhibiting peripheral immunosuppression (e.g., a non-selective β-blocker to protect lymphocyte function) with a drug that can penetrate the BBB to target central neuroinflammation (e.g., an NLRP3 inflammasome inhibitor) during the acute phase. The advantage of this strategy is that it concurrently addresses the core paradox created by circuitry dysregulation: “central hyper-inflammation” and “peripheral immunosuppression”. Furthermore, Vertical Integration should not be overlooked, which involves intervening at different levels of the same pathological axis. For example, combining a drug that modulates higher centers like the hypothalamus with a drug that acts directly on peripheral immune organs (such as the spleen) could reset the function of the entire regulatory axis from the top down (142).

The post-stroke immune response is not a static process but a dynamic evolution with distinct phases. Ignoring this time-dependency is likely a key reason for the failure of many past large-scale anti-inflammatory clinical trials. Applying an intervention at the wrong time point is not only ineffective but can even be harmful. Therefore, future therapeutic strategies must be temporally-dependent. The hyperacute/acute phase (hours to days) is characterized by intense neuroinflammation, cell death, and BBB disruption. The primary therapeutic goal is therefore to inhibit the excessive inflammatory cascade and mitigate secondary brain injury. During this window, strategies targeting myeloid cell overactivation (e.g., NLRP3 inhibitors) or the early cytokine storm have the strongest theoretical rationale. In the subacute/chronic phase (days to weeks), the core mission of the immune system shifts from “attack” to “clean-up and reconstruction”. At this stage, certain functions of immune cells, especially M2-like macrophages and regulatory T cells, such as phagocytosing cellular debris, secreting neurotrophic factors, and promoting angiogenesis, are essential and beneficial for tissue repair and neuroplasticity. Studies have found that CCR2-dependent monocytes/macrophages exacerbate acute brain injury after ischemic stroke in mice but are beneficial for long-term functional recovery (143). Therefore, during this window, the therapeutic goal should correspondingly shift to actively promoting the orderly “pro-resolution” of inflammation and fostering a pro-reparative microenvironment. At this point, continuing the use of potent, broad-spectrum immunosuppressants could be counterproductive, whereas strategies aimed at enhancing Treg function or promoting M2 polarization hold greater potential (88).

## 7 Conclusion and future perspectives

IS is far from being an isolated injury event confined to the CNS, it is a systemic disease that triggers a complex, dynamic, and enduring whole-body response (16). In this review, we have systematically proposed a theoretical framework of a Neuroimmune Regulatory Circuitry. This sophisticated network, which maintains organismal homeostasis under physiological conditions, undergoes a catastrophic functional collapse at multiple levels—afferent perception, central integration, and efferent response—following an IS event. The strength of this framework lies in its ability to unify the seemingly contradictory pathological phenomena observed after IS, including intense inflammation within the brain, profound peripheral immunosuppression, emergency hematopoiesis in the bone marrow, and long-term multi-organ complications, under a single, coherent pathophysiological model with intrinsic causal logic (144). Therefore, reconceptualizing stroke as a disease of Neuroimmune Regulatory Circuitry dysregulation is not only a more profound understanding of its complexity but, more importantly, provides a new and more rational theoretical foundation for identifying novel biomarkers and developing multi-target, multi-stage innovative therapeutic strategies.

However, we still face numerous challenges in translating this theory into effective clinical practice. Foremost among them is patient heterogeneity (145). Current IS immunology research often treats patients as a homogenous group, which is far from the clinical reality. A patient’s age, sex, and underlying comorbidities (such as diabetes) can themselves cause baseline dysregulation of the neuroimmune circuitry. Therefore, they are all critical variables that determine the pattern of the post-stroke immune response (146–148). Future research, especially clinical trial design, can leverage combinations of biomarkers (e.g., NLR, HRV, cytokine profiles) to perform precise immuno-phenotyping of patients before treatment. This would identify those in states of extreme immunosuppression or hyper-inflammation, which is the logical prerequisite for achieving personalized immunomodulatory therapy.

Among the many factors contributing to patient heterogeneity, as discussed in the chapter on the physiological circuitry, the gut and its vast symbiotic microbiome is a potent and modifiable variable influencing the function of the Neuroimmune Regulatory Circuitry (149). Consequently, a core frontier for future research is to elucidate how stroke-induced dysbiosis mechanistically exacerbates the dysregulation of the neuroimmune circuit. Based on this, targeting the gut micro-ecology—through probiotics, dietary interventions, or even fecal microbiota transplantation (FMT)—has emerged as a highly attractive new therapeutic direction. Its goal is not only to restore gut health but to serve as a systemic strategy to recalibrate the dysregulated Neuroimmune Regulatory Circuitry, thereby improving patients’ clinical outcomes.

As we shift our focus from systemic interventions like the gut to the molecular level of drug development, traditional anti-inflammatory strategies also face a conceptual revolution. For a long time, immunotherapy has focused on inhibiting pro-inflammatory pathways. However, this is often a double-edged sword, as it may simultaneously suppress the necessary reparative functions of the immune system. A more advanced and physiologically attuned concept is to shift from anti-inflammation to pro-resolution. The physiological resolution of inflammation is not a passive process but is actively coordinated by a class of endogenous lipid molecules known as Specialized Pro-resolving Mediators (SPMs), such as resolvins, protectins, and maresins (150). By activating specific receptors, these molecules not only inhibit inflammation but also actively promote the clearance of dead cells, suppress pain, and activate tissue regeneration programs. Therefore, developing drugs that can mimic or enhance the function of endogenous SPMs represents an exceptionally elegant and promising direction for the future of stroke immunotherapy (151).

Whether elucidating the complex role of the gut microbiota or developing sophisticated pro-resolution therapies like SPMs, the realization of these goals will greatly benefit from the impetus of cutting-edge technologies. The in-depth analysis of the complex system of the Neuroimmune Regulatory Circuitry is entering a new era led by technological innovation. Single-cell and spatial transcriptomics will allow us to map the dynamic changes of every cell subpopulation in the post-stroke brain and immune organs with unprecedented resolution (152). High-dimensional cytometry can help us identify novel immune cell phenotypes in clinical samples that are correlated with prognosis (153). Advanced viral tracing and opto/chemogenetics will permit us to map and manipulate specific neural circuits connecting the central and peripheral systems with higher precision in preclinical models (154, 155). And novel PET molecular imaging probes hold the promise of enabling non-invasive, dynamic, and visual tracking of neuroinflammation and specific immune cell infiltration in the brain in the future (156).

Finally, the ultimate challenge that perhaps integrates all of the above is the time dimension of the immune response. The post-stroke immune reaction has distinct temporal characteristics, and cutting-edge technologies are the key tools for us to precisely delineate this timeline. The core challenge for future clinical translational research will be to precisely define the optimal therapeutic window for different immune interventions. Inhibiting injury in the hyperacute phase versus promoting repair in the subacute/chronic phase requires distinctly different therapeutic rationales and drugs. Elucidating the key molecular switches that drive this phased transition and developing therapeutic regimens that can be dynamically adjusted to the patient’s pathological stage will be the key to whether stroke immunotherapy can ultimately succeed. Therefore, mastering and leveraging this temporal dimension of the immune response will be the central task for future research in this field.
